# Foliar application of magnesium and amino acids to off-season maize: a strategy to improve photosynthetic and antioxidant metabolism

**DOI:** 10.3389/fpls.2026.1850949

**Published:** 2026-07-01

**Authors:** Gabriel Oliveira Neves, Sirlene Lopes Oliveira, Israel Alves Filho, Tatiani Mayara Galeriani, João William Bossolani, José Roberto Portugal, Luiz Gustavo Moretti, Carlos Alexandre Costa Crusciol

**Affiliations:** 1 School of Agricultural Sciences, São Paulo State University (UNESP), Botucatu, Brazil; 2 Federal Institute of Education, Science and Technology of Mato Grosso (IFMT), Sorriso, Brazil; 3 Embrapa Cerrados, Planaltina, Federal District, Brazil

**Keywords:** abiotic stress, foliar application, plant physiology, stimulating nutrition, *Zea mays* L.

## Abstract

**Introduction:**

In Brazil, maize is commonly cultivated during the off-season (fall/winter), a period characterized by reduced rainfall that substantially limits crop productivity. Foliar application of nutrients and amino acids has emerged as a promising strategy to mitigate physiological damage caused by water deficit by enhancing plant tolerance to abiotic stress. However, the combined effects of magnesium (Mg) and amino acids (AA) on off-season maize have not yet been investigated.

**Methods:**

A field experiment was conducted to evaluate the effects of foliar applications of Mg and AA on the photosynthetic and antioxidant metabolism of off-season maize. Four treatments were evaluated: (I) control, (II) foliar application of Mg, (III) foliar application of AA, and (IV) combined foliar application of Mg and AA (Mg+AA). Leaf nutritional status, photosynthetic traits, antioxidant enzyme activity, and grain yield were assessed.

**Results:**

The combined Mg+AA treatment improved leaf nutritional status, photosynthetic performance, enzymatic activity, and antioxidant metabolism compared with the isolated applications of Mg or AA. Grain yield increased by approximately 15% under Mg+AA relative to the control, whereas isolated applications of AA and Mg increased yield by 10% and 5%, respectively.

**Discussion:**

The results demonstrate additive effects of Mg and AA on the photosynthetic and antioxidant metabolism of maize plants. The combined foliar application of Mg and AA effectively alleviated stress effects and enhanced grain yield, representing a promising strategy to improve the productivity of off-season maize under field conditions.

## Graphical Abstract

**Figure:**
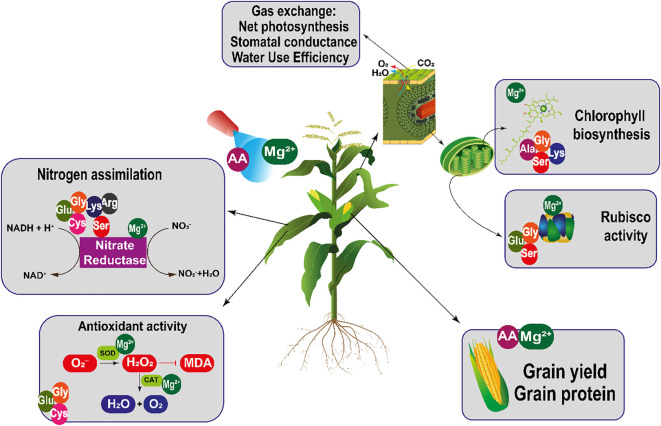
The combined application of magnesium (Mg) and amino acids (AA) had a additive effect on nutrient assimilation, particularly nitrogen. This was achieved by enhancing the activity of the nitrate reductase enzyme. Photosynthesis also increased, driven by elevated chlorophyll content, improved gas exchange, and greater Rubisco activity. Additionally increased antioxidant enzyme activity reduced the reactive oxygen species (ROS). Consequently, maize plants treated with Mg and AA exhibited higher protein content in the grains and greater grain yield. Ala, Alanine; Arg, Arginine; Cys, Cysteine; Glu, Glutamic Acid; Gly, Glycine; Lys, Lysine; Ser, Serine.

## 1 Introduction

Brazil is one of the world’s top producers of maize (*Zea mays* L.), primarily due to the production of a second crop (off-season maize) after the summer soybean harvest. This soybean-maize succession system has significantly enhanced the national grain supply while optimizing the use of agricultural lands (Nóia Júnior and Sentelhas, 2020). Currently, off-season maize surpasses summer maize in both area cultivated and production and accounts for 77% of Brazilian maize production (Nóia Júnior and Sentelhas, 2019; Conab, 2023). Furthermore, data from the past five years (2019 to 2024) indicate that the national grain yield of off-season maize (5238 kg ha^−1^) is nearing that of summer maize (5839 kg ha^−1^) (Conab, 2024). Summer maize benefits from more favorable climatic conditions, particularly optimal rainfall, which supports better plant development.; however, the use of technologies and adequate management have promoted an increase in the productivity of off-season maize (Nóia Júnior and Sentelhas, 2019). Despite this, unfavorable environmental conditions, such as drought and heat waves, reduce production and lead to economic losses. Climate change is intensifying the negative effects of these abiotic stresses on agriculture and represents a global challenge for food production (Hura et al., 2022; Kumari et al., 2022).

Drought triggers several physiological and biochemical disturbances in plants, primarily affecting water transport and usage (Seleiman et al., 2021). One of the most significant consequences is an imbalance in the production of reactive oxygen species (ROS). Under these conditions, the excessive generation of ROS disrupts photosynthetic activity and leads to the degradation of cellular components, which adversely impacts plant growth and crop yield (Impa et al., 2012). To mitigate the damage caused by drought, it is crucial to enhance stress tolerance mechanisms that involve the production of antioxidant enzymes. These enzymes play a vital role in maintaining a proper balance between the production and scavenging of ROS (Kaur and Asthir, 2017). Consequently, biostimulants have emerged as a promising strategy to alleviate the harmful effects of abiotic stress, in addition to increasing nutrient use efficiency, photosynthetic activity and crop productivity (Bhupenchandra et al., 2022).

Amino acids (AAs) are a category of plant biostimulants that are gaining increasing attention for agricultural crop management. AA synthesis by plants requires large amounts of energy; thus, the application of readily absorbable AA, especially at critical stages in the plant life cycle, can accelerate the developmental process by spending less energy (Popko et al., 2018). AA hold potential as stress-mitigating agents, organic nitrogen (N) sources, and precursors for plant hormones (Kadotani et al., 2016; Batista‐Silva et al., 2019; Dellero, 2020). Amino acids are involved in several physiological and metabolic processes associated with plant growth and stress responses, including chlorophyll synthesis, hormonal regulation, maintenance of water balance, and nitrogen metabolism (Sowmya et al., 2023). Therefore, these compounds may contribute to improved plant performance under adverse environmental conditions by supporting physiological and biochemical adjustments. Teixeira et al. (2018) demonstrated an increase in nitrate, amino acids and total nitrogen content in soybean leaves, in addition to an increase in grain yield under foliar application of a biostimulant based on AAs (cysteine, phenylalanine, glycine and glutamate). Nonetheless, its exogenous application of AA is a relatively new approach, and many aspects of the effects on the physiology of cultivated plants under field conditions are poorly understood.

Conversely, fertilizers are inputs widely studied and used in agriculture but are not considered a category of biostimulant (du Jardin, 2015), however, several studies have suggested that the foliar application of nutrients in well-nourished plants has a stimulating role in plant metabolism, which has been promising in reducing metabolic damage under drought conditions and enhancing the productive potential of agricultural crops (Rosolem, 2002; Rodrigues et al., 2021a; Galeriani et al., 2022; Kumari et al., 2022; Oliveira et al., 2022).

In a previous study we demonstrated that foliar application of magnesium (Mg) in small doses to well-nourished plants efficiently improves photosynthetic activity, antioxidant metabolism by increasing the activity of the enzymes superoxide dismutase, catalase and ascorbate peroxidase leading to increased productivity of soybean and maize plants (Rodrigues et al., 2021a), due to Mg’s role in chlorophyll synthesis, assimilate transport and utilization, enzyme activation, and protein synthesis (Cakmak, 2013; Ishfaq et al., 2022).

Generally, studies based on biostimulants are focused on their isolated effects on plant metabolism; however, the adoption of two or more strategies may be a key factor in obtaining more adapted and photosynthetically efficient plants under abiotic stress conditions. In this regard, Simultaneous application of biostimulants often has additive effects that further enhance plant growth and development (Zamljen et al., 2023). The application of macronutrients associated with AA was efficient in mitigating the physiological impacts of water deficit and improving quality traits in sugarcane (Jacomassi et al., 2024). However, despite the recognized physiological roles of Mg and amino acids, information regarding their combined foliar application under field conditions remains scarce, particularly for off-season maize cultivated under rainfed conditions. Therefore, we hypothesized that the combined application of Mg and amino acids would enhance physiological, antioxidant, and productive responses compared with their isolated application. Thus, the objective of this study was to evaluate the effects of combined foliar Mg and amino acid application on the photosynthetic, antioxidant, and productive metabolism of off-season maize.

## 2 Materials and methods

### 2.1 Characteristics of the experimental site

The experiment was conducted during the autumn/winter seasons of 2021 and 2022. The experimental site is part of the Experimental Lageado area of São Paulo State University (UNESP) in the municipality of Botucatu, São Paulo, Brazil (48° 26’ W, 22° 51’ S). The municipality is located at an altitude of 786 m above sea level and has a Cwa-type climate, which is mesothermal with summer rainfall and dry winters according to Köppen’s classification. The average annual precipitation is 1360 mm, and the average annual temperature is 20.7 °C (UNICAMP, 2020). The soil in the cultivation area is a Dystroferric Red Latosol (Santos et al., 2018) which corresponds to oxisols (Soil Survey Staff, 2014).

Maize was cultivated under rainfed conditions. To illustrate the climatic conditions during the cropping period, a water balance was constructed using spreadsheets developed by Rolim et al. (1998) and using the method proposed by Thornthwaite and Mather (1995). Climatic data, including rainfall and maximum and minimum temperatures from a nearby meteorological station, were entered into the spreadsheet along with soil water-holding capacity (CAD) data (Batista‐Silva et al., 2019), latitude, and insolation.

The experimental area is annually cultivated under a soybean–maize crop rotation system. The chemical and physical characteristics of the soil in the 0.0–0.2 m layer were determined (Van Raij et al., 2001) prior to the installation of the experiment and are presented in **Supplementary Table 1**.

### 2.2 Maize cultivation

Off-season maize was cultivated between the months of March and August in 2021 and 2022. Seeds of maize hybrid FS587 (Forseed^®^) were treated with fungicides (carboxin + thiram, 1 g of active ingredient per kg of seeds) and sown at 3.1 seeds m^-^¹ with 0.45 m row spacing, targeting an initial population of ~68,888 plants ha^-^¹. Fertilization at sowing was performed by applying 300 kg ha^−1^ of formulated 08-28-16 (N-P_2_O_5_-K_2_O) in both years (van Raij et al., 1998). At the V_5_ growth stage, 100 kg ha^−1^ of N was applied as urea as topdressing fertilization. Other phytosanitary treatments were performed according to recommendations for maize cultivation (Galvão et al., 2017).

### 2.3 Experimental design and treatments

The experiment was conducted in a randomized complete block design consisting of four foliar application treatments with eight replications (8 blocks in each growing season) in two cultivation seasons: (I) control, (II) Mg, (III) AA, and (IV) Mg+AA. The Mg product was applied at a dose of 500 g ha^−1^ of Mg (MgCl_2_), and the AA-based product was applied at a dose of 1250 g ha^−1^ along with a silicone-based adjuvant (30 mL ha^−1^; Disperse^®^; Ubyfol; Uberaba, Brazil) was included in all spray solutions, including the control treatment, to ensure uniform application conditions and to isolate the physiological effects of Mg and AA from any potential adjuvant-related effects. The composition of the AA product is described in **Table 1**. The doses were defined based on the recommendations of the respective manufacturers. Both products were applied at the Vt development stage. This stage represents the transition from the vegetative stage to the reproductive stage and corresponds to the appearance of the tassel. This is a critical period for the reproductive growth, and any environmental stress during this period can significantly reduce yield potential (Galvão et al., 2017). Foliar spraying was carried out in the late afternoon using a pressurized backpack sprayer with CO_2_ at a constant pressure of 1.8 bar and a spray boom containing 6 Teejet nozzles (model XR 110–02 VP), which generated a spray volume of 200 L ha^−1^. Foliar spraying was performed out according to the technical recommendations of the respective manufacturers and was done on the same day so that the application conditions were the same for all treatments. Each experimental unit consisted of plots with ten rows, each row measuring 10 meters in length, resulting in a total plot area of 45 m². The useful area for data collection within each plot was 15 m².

**Table 1:** Dose and composition of the amino acid-based product applied to maize leaves.

Dose	Composition (g ha^−1^)									
	Ala	Arg	Asp	Cys	Glu	Gly	His	Hyp	Ile	Leu
1250 g ha^−1^	212.5	0.075	200	0.525	0.788	0.1625	6.25	0.0625	0.05	0.075
	Lys	Met	Phe	Pro	Ser	Hyl	Thr	Trp	Tyr	Val
	0.05	0.025	0.05	96	0.05	48	0.05	0.05	0.025	0.10

Ala, Alanine; Arg, Arginine; Asp, Aspartic acid; Cys, Cysteine; Glu, Glutamic Acid; Gly, Glycine; His, Histidine; Hyp, Hydroxyproline; Ile, Isoleucine; Leu, Leucine; Lys, Lysine; Met, Methionine; Phe, Phenylalanine; Pro, Proline; Ser, Serine; Hyl, Hydroxylysine; Thr, Threonine; Trp, Tryptophan; Tyr, Tyrosine; Val, Valine.

### 2.4 Plant sampling and analysis

Chemical and physiological analyses were conducted at the R_1_ phenological stage (Ritchie et al., 1993). Samples were taken from the middle third of the leaves of 10 competitive plants in each experimental plot. For nutritional analysis, leaves were oven-dried at 60 °C to a constant weight and ground (Malavolta et al., 1997). SPAD index and gas exchange were measured between 8:00 and 10:00 a.m. Samples for photosynthetic pigments, nitrate reductase, and Rubisco activities were collected in Falcon tubes, transported in liquid nitrogen, and stored at -80 °C until analysis.

#### 2.4.1 Leaf nutrient analysis

To determine the N content, 0.5 g of dry and ground leaves were subjected to the semi-micro Kjeldahl method, according to Kirk (1950). The concentrations of P, K, Ca, Mg, S, Fe, Mn, B, Cu, and Zn were obtained after nitric–perchloric digestion and quantified by atomic absorption spectrophotometry (Malavolta et al., 1997).

#### 2.4.2 Photosynthetic analysis

The SPAD index was determined by non-destructive analysis using a SPAD 502 chlorophyll meter (Konica Minolta^®^). To measure photosynthetic pigments (Chl *a*, Chl *b*, and carotenoids), three 0.5-cm discs were cut from the middle third of the leaf using a paper punch and the extract was obtained by maceration in acetone (80% v/v.) (Lichtenthaler, 1987). The concentrations of Chl *a*, Chl *b*, and carotenoids were determined by measuring the absorbance 664, 647, and 480 nm, respectively, using a spectrophotometer. Photosynthetic pigment concentrations were calculated based on the methods described by Wellburn (1994).

Gas-exchange parameters were assessed using a portable infrared gas analyzer (LI-6400, LI-COR Biosciences Inc., Lincoln, NE, USA), configured with atmospheric CO_2_ concentration ranging from 380 to 400 μmol mol^-^¹, photosynthetically active radiation (PAR) set at 1100 μmol quanta m^-^² s^-^¹ using LED lamps, leaf chamber temperature maintained at 25–27 °C, and relative humidity controlled at 60%–70%. The following parameters were measured: net photosynthetic rate (*A*, μmol CO_2_ m^-^² s^-^¹), stomatal conductance (*gs*, mol H_2_O m^-^² s^-^¹), internal CO_2_ concentration (*Ci*, μmol mol^-^¹), transpiration rate (*E*, mmol H_2_O m^-^² s^-^¹), water use efficiency [WUE, μmol CO_2_ (mmol H_2_O)^-^¹], and carboxylation efficiency (A/Ci).

#### 2.4.3 Enzymatic activity

To determine the leaf content of Nitrate Reductase (NR), frozen leaves were macerated in liquid nitrogen and added to extraction buffer containing 25 mM Tris-HCl (pH 8.5), 1 M EDTA, 1 mM DTT, 1% BSA, 20 μM FAD, and 200 μM leupeptin. After centrifugation and filtration, aliquots of the supernatant were added to microtubes containing a MgCl_2_-based reaction buffer. The extract was incubated, and the reaction was stopped by the addition of zinc acetate. The supernatant was centrifuged again, and the formation of nitrite was determined using a colorimetric technique (Hageman and Reed, 1980).

Rubisco activity was determined according to the method described by Degl’Innocenti et al. (2002), in which the frozen leaves were macerated with a pestle pre-frozen with liquid nitrogen. Then, a buffer containing 50 mM Tris-HCl (pH 7.8), 2 mM Na_2_EDTA, 5 mM β-mercaptoethanol,1 mM PMSF was added to 3 cm^3^ of the leaf sample. The solution was centrifuged at 12000xg for 10 min. The supernatant was used to prepare the reaction mixture, which was prepared using 50 mM Tris-HCl (pH 7.8), 10 mM MgCl_2_, 30 mM NaHCO_3_, 5 mM β-mercaptoethanol, 83 nkat PGK (E.C. 2.7.2.3), 83 nkat GAPDH, 17 nkat glycerol-3-phosphate isomerase (E.C. 1.1.1.8), 100 nkat triose phosphate isomerase (E.C. 5.3.1.1), 0.15 mM NADH, and 0.5 mM ATP. Aliquots of the reaction mixture were analyzed spectrophotometrically by monitoring the rate of NADH oxidation at a wavelength of 340 nm (Reid et al., 1997). Specifically, Rubisco activity was determined from the difference in absorbance readings at 0 and 1 min (without removing the cuvette from the spectrophotometer), and the results were expressed in μmol min^−1^ mg protein^−1^.

#### 2.4.4 Reactive oxygen species and antioxidant enzymes

The determination of lipid peroxidation – malondialdehyde (MDA) – and hydrogen peroxide (H_2_O_2_) were performed by macerating 0.4 g sample of frozen leaf in liquid nitrogen. MDA determination was performed using the thiobarbituric acid (TBA) reaction method. After collecting and grinding the leaves, the tissue was homogenized in a NaCl solution (175 mM) and Tris-Cl buffer (50 mM, pH 8.0), followed by centrifugation to obtain the supernatant. An aliquot of the supernatant was mixed with TBA solution (0.5% in 20% trichloroacetic acid) and heated at 95 °C for 25 minutes. After centrifugation, absorbance was measured at 532 nm, with correction for turbidity at 600 nm. MDA concentration was calculated using the molar extinction coefficient of 155 mM^-^¹ cm^-^¹, and the results were expressed in nmol of MDA per gram of fresh weight (Heath and Packer, 1968). To determine the H_2_O_2_ concentration, after extraction of the leaf tissue with 0.1% trichloroacetic acid (TCA), the supernatant was mixed with potassium phosphate buffer (100 mM, pH 7.0) and reagent containing KI (1% m/v in distilled water). The reaction was kept in the dark for 1 hour, and the absorbance was measured at 390 nm. A standard curve with known concentrations of H_2_O_2_ was used to calculate the amount of hydrogen peroxide in the samples. The method proved to be efficient and reliable for the quantification of H_2_O_2_, allowing the evaluation of oxidative stress in corn leaves (Alexieva et al., 2001).

For determination of superoxide dismutase (SOD, EC: 1.15.1.1), 50 µL sample of the plant extract were homogenized in potassium phosphate buffer (0.1 M, pH 7.8) containing EDTA (0.1 mM). The homogenate was centrifuged at 13,000 g for 10 min, and the supernatant (crude SOD extract) was used for enzymatic assays. SOD activity was determined by a photometric method using a reaction system containing methionine, riboflavin, and nitroblue tetrazolium (NBT). Absorbance was measured at 560 nm, and SOD activity was calculated based on the inhibition of NBT reduction. The results were expressed as units of enzyme activity per milligram of protein (Giannopolitis and Ries, 1977). For the determination of catalase activity (CAT, EC: 1.11.1.6), a 5 µL sample of the plant extract was combined with 3 mL of buffer A, which consisted of 100 mL of 100 mM potassium phosphate buffer (pH 7.5) plus 250 µL of 30% hydrogen peroxide (H_2_O_2_). The reaction was monitored for one minute at 25 °C using a quartz cuvette, and the degradation of H_2_O_2_ was measured by recording the absorbance at 240 nm with a UV-Vis spectrophotometer. Results were expressed as µmol min^-^¹ mg^-^¹ of protein (Reis et al., 2015).

#### 2.4.5 Yield and quality components

At physiological maturity, plant height (PH, cm) was measured in 10 plants from the central area of each plot, excluding border rows. Subsequently, plants from a 15 m² net plot area were harvested, and 10 representative ears were selected for analysis. The number of rows per ear (NRE), the number of grains per row (NGR), and the total number of grains per ear (NGE) were recorded, with NGE calculated by multiplying NRE by NGR. hundred-grain weight (W100G, g) was measured in triplicate for each plot. Grain yield (GY) was determined following the threshing of the harvested ears from the useful area. The yield was calculated based on the total weight obtained, adjusted for a moisture content of 13%. Finally, GY was expressed kg ha^-^¹.

#### 2.4.6 Protein content in grain

Specific proteins were extracted from 0.1 g of ground maize flour as described by Chen et al. (2018) using a 1.0-ml volume of the following solvents: 10 mM Tris-HCl buffer (pH 7.5) for albumin; 1 M NaCl for globulin; 60% n-propanol containing 1 mM EDTA-2Na for prolamin; or 0.05 M NaOH for glutelin. The mixture was shaken for 2 h at room temperature, and the extracts were separated from the residues by centrifugation for 15 min at 12,000 rpm and 4 °C. This procedure was repeated three times, and the extracts were stored at -20 °C until further analysis. The quantity of each protein was determined using the Coomassie brilliant blue G-250 dye-binding method with bovine serum albumin as the standard (Bradford, 1976). Quantitative analysis was performed using an Infinite M200 microplate reader (Tecan Group, Männedorf, Switzerland) as described by Peng et al. (2014).

### 2.5 Statistical analysis

Initially, data normality and homoscedasticity were assessed using the Shapiro–Wilk test (Shapiro and Wilk, 1965) and Levene’s test (Levene, 1960). After confirming that both assumptions for parametric tests were met, a factorial analysis of variance (ANOVA) was performed using the F-test, considering crop seasons (years) as random factors and foliar applications (treatments) as fixed factors. The interactions between years x treatments were not significant (p > 0.05) for any of the analyzed variables (**Supplementary Tables 2**, **5**). This indicates that the effect of foliar applications on the measured variables did not differ significantly between the two crop seasons. Therefore, the means of the two seasons were combined to simplify the analysis and improve the robustness of the results. Finally, the means of the foliar application treatments were compared using the least significant difference (LSD) test at a significance level of p ≤ 0.05. The LSD test is efficient in detecting real differences between treatments under field conditions, as long as the assumptions of normality and homogeneity of variances are met, and its effectiveness is even greater when used in experiments containing few treatments (Agbangba et al., 2024), as in the present study.

Principal component analysis (PCA) was performed to explore the multivariate relationships among physiological, biochemical, nutritional, and yield variables and to visualize treatment discrimination. Prior to analysis, variables were standardized (mean = 0, standard deviation = 1) to eliminate scale effects. PCA was conducted using the FactoMineR package, and graphical visualization was generated using the factoextra package in R software (R Core Team, 2026). Confidence ellipses (95%) were included to illustrate the dispersion and clustering of treatments in the multivariate space.

## 3 Results

### 3.1 Hydroclimatic balance

**Figure 1:**
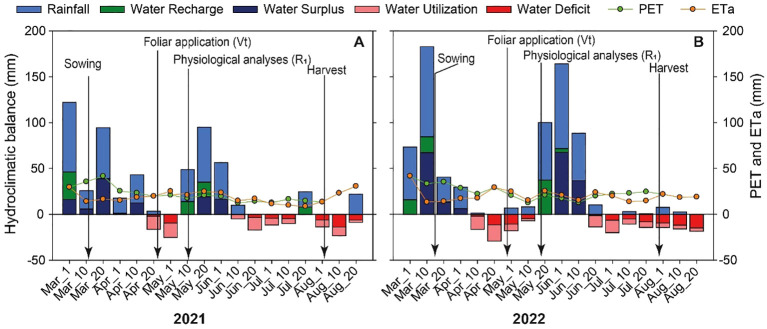
Hydroclimatic balance during the off-season maize season in **(A)** 2021 and **(B)** 2022 in Botucatu, São Paulo state. PET, Potential evapotranspiration, Eta, actual evapotranspiration.

The hydroclimatic balance proposed by Thornthwaite and Mather (1995) helps to monitor soil water and functions as a valuable tool for strategic agricultural planning and water resource management. In both crop years, maize was sown during a period of water surplus, which occurs when rainfall exceeds the demand for potential evapotranspiration (ETP) (Passos et al., 2017). During this time, positive water balances are utilized to recharge the soil until it reaches its maximum storage capacity (**Figure 1**). Under these circumstances, the soil theoretically maintained adequate humidity for the development of maize plants. Additionally, another surplus period was observed during the flowering stage in both 2021 and 2022 crop years.

However, there were prolonged periods of water deficit in both cultivation seasons during which water utilization exceeded the water recharge from rainfall, resulting in insufficient soil moisture to meet crop demands. Water restriction was more pronounced in the 2021 season; the accumulated precipitation during cultivation was 288 mm, with water deficits between the VT and R_1_ phenological stages and between R_3_ and R_5_ (**Figure 1A**). In the 2022 season, the distribution of rainfall was similar to that in the previous year, but the accumulated precipitation was 386 mm. In the second season, periods of water deficit occurred between developmental stages V_8_ and Vt and between reproductive stages R_3_ and R_6_ (**Figure 1B**).

### 3.2 Leaf nutritional status and nitrate reductase activity

**Figure 2:**
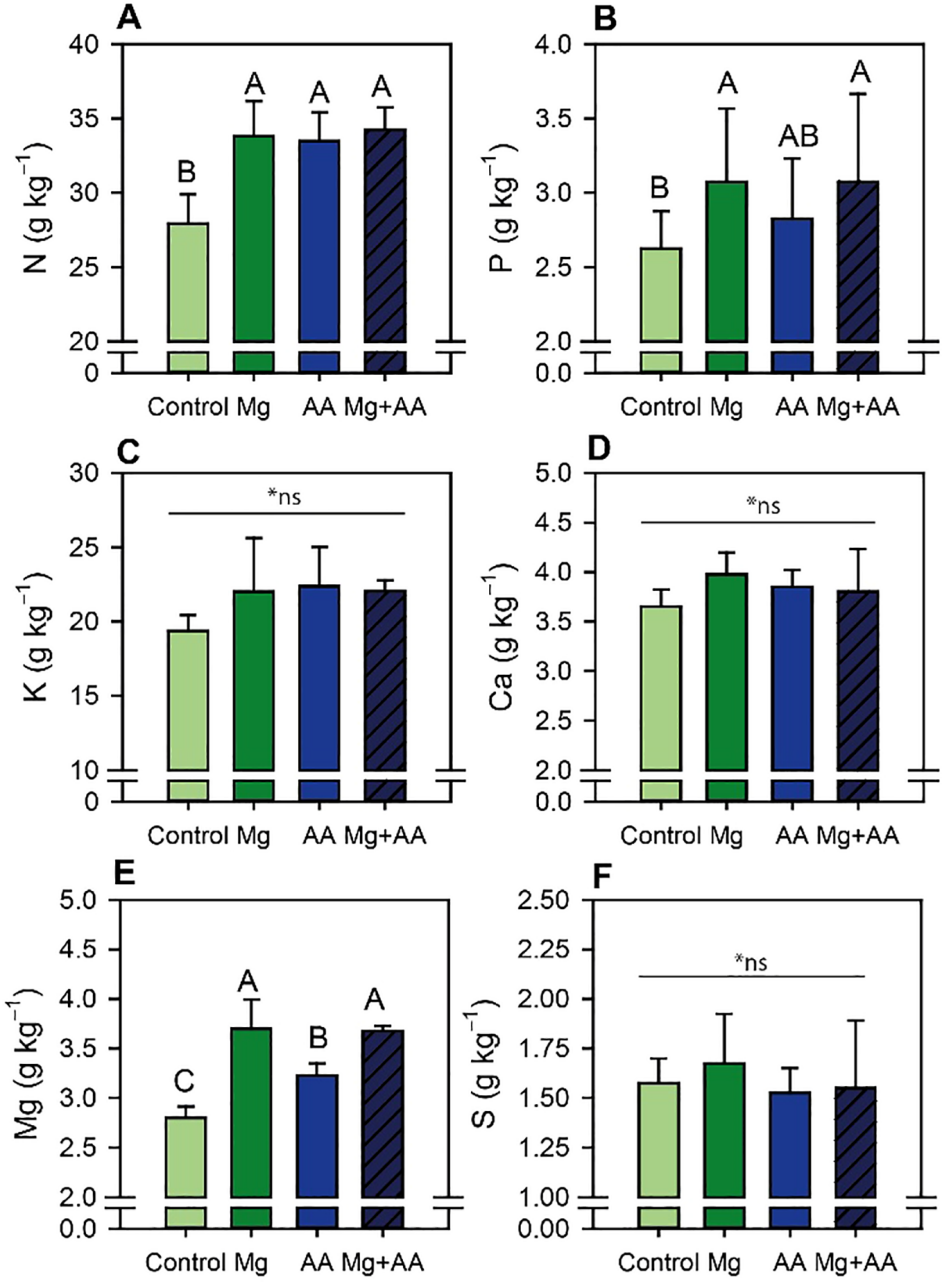
Leaf macronutrient levels in maize plants as affected by the foliar application of magnesium (Mg) and/or amino acids (AA): **(A)** N, **(B)** P, **(C)** K, **(D)** Ca, **(e)** Mg, and **(f)** (S) Bars represent means across the 2021 and 2022 off-season growing periods. Different letters indicate significant differences among treatments (LSD, p ≤ 0.05). ns, not significant. Error bars represent standard deviation (n = 8). *ns, not significant.

Foliar application of Mg and/or AA increased leaf N, Mg, and P concentrations, whereas micronutrient concentrations were not affected by the treatments (**Figure 2**; **Supplementary Table 2**). The strongest nutritional response was consistently observed under Mg+AA, which increased leaf N and Mg concentrations by 23% and 31%, respectively, relative to the control. Nitrogen accumulation increased under all foliar applications, while the largest increases in Mg concentration were observed under Mg-containing treatments. In contrast, P concentration responded only to Mg and Mg+AA applications. Leaf nutrient concentrations were generally higher in 2021, likely associated with the more favorable rainfall distribution during vegetative growth despite the lower cumulative precipitation relative to 2022 (**Figure 1**).

### 3.3 SPAD index and photosynthetic pigments

**Figure 3:**
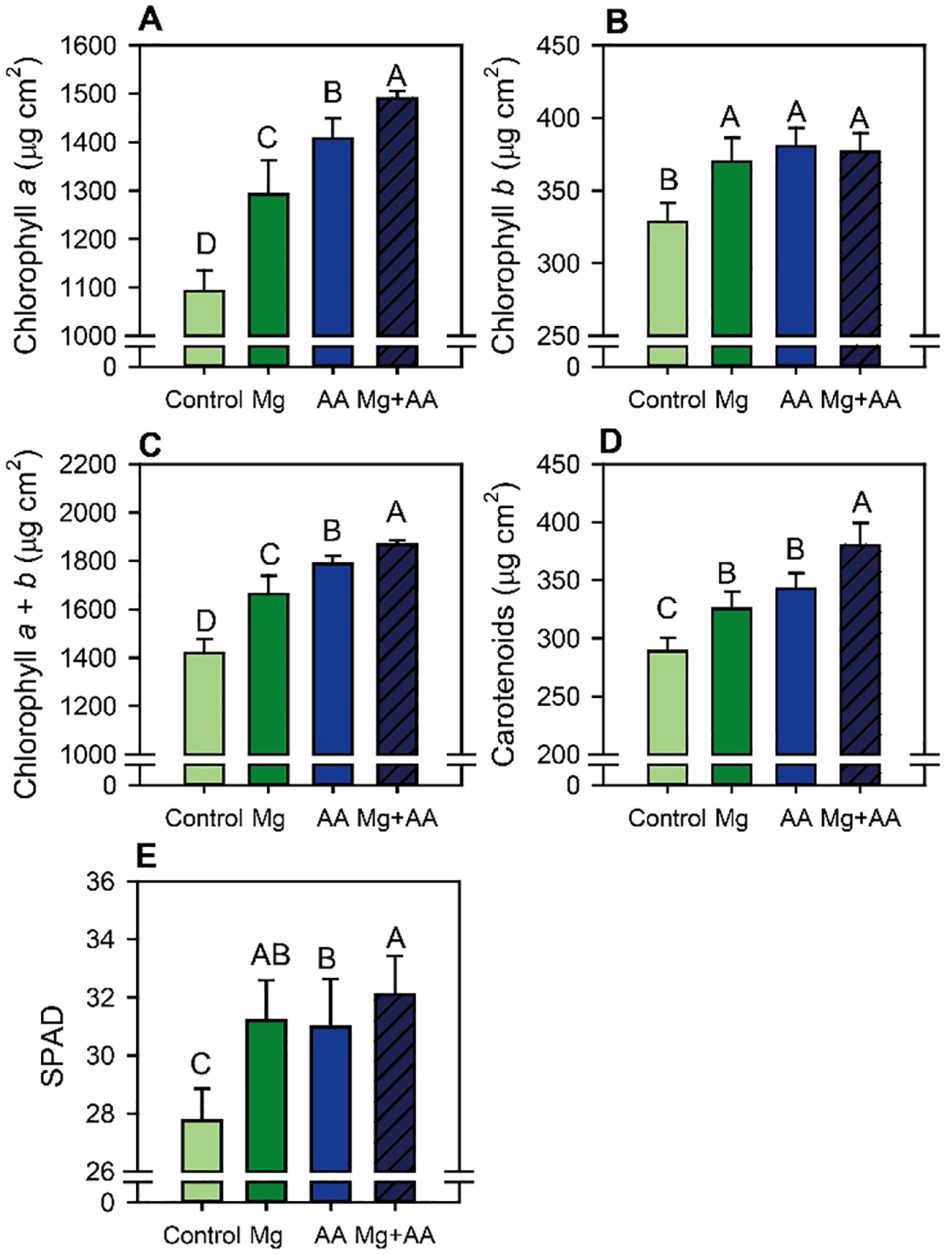
Contents of **(A)** chlorophyll *a* (Chl *a*), **(B)** chlorophyll *b* (Chl *b*), **(C)** chlorophyll *a*+*b* (Chl *a*+*b*), **(D)** carotenoids and **(E)** SPAD index in the leaves of maize plants as affected by the foliar application of magnesium (Mg) and/or amino acids (AA). Bars represent means across the 2021 and 2022 off-season growing periods. Different letters indicate significant differences among treatments (LSD, p ≤ 0.05). Error bars represent standard deviation (n = 8).

Foliar treatments increased chlorophyll concentrations, carotenoid content, and SPAD values relative to the control (**Figure 3**). Overall, responses followed the order Mg+AA > AA > Mg, with the combined treatment promoting the greatest increases in chlorophyll a, total chlorophyll concentration, and carotenoid accumulation. In contrast, chlorophyll b exhibited smaller differences among treatments. As a result, total chlorophyll concentration increased by 31% under Mg+AA, whereas AA and Mg resulted in intermediate gains of 26% and 17%, respectively. Carotenoid concentration and SPAD values exhibited a similar response pattern across treatments.

### 3.4 Gas exchange

**Figure 4:**
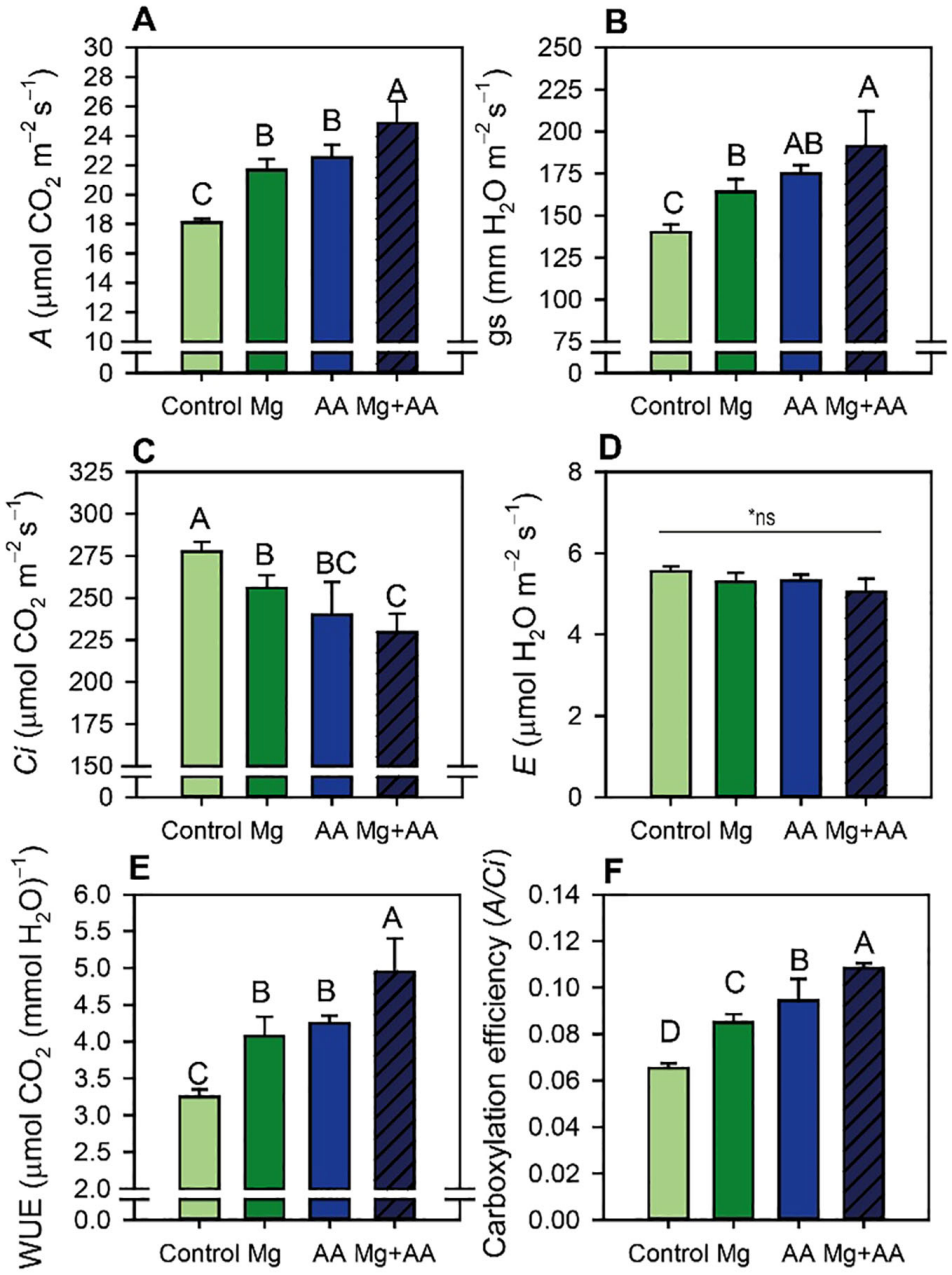
Photosynthetic parameters in maize leaves as affected by the foliar application of magnesium (Mg) and/or amino acids (AA): **(A)** net photosynthesis (A), **(B)** stomatal conductance (gs), **(C)** CO_2_ concentration in the stomatal chamber (Ci), **(D)** leaf transpiration (E), **(E)** water use efficiency (WUE), and **(F)** carboxylation efficiency (*A/Ci*). Bars represent means across the 2021 and 2022 off-season growing periods. Different letters indicate significant differences among treatments (LSD, p ≤ 0.05). ns, not significant. Error bars represent standard deviation (n = 8). *ns, not significant.

In general, foliar applications improved gas exchange results, with the treatment effects following the order Mg+AA > AA > Mg in all parameters (**Figure 4**). The combined Mg+AA treatment promoted the greatest increase in net photosynthesis (A; 37%), accompanied by the greatest increase in stomatal conductance (*gs*; 36%) and the most pronounced reduction in intercellular CO_2_ concentration (*Ci*; 17%), which may indicate a response pattern suggestive of higher CO_2_ demand in the mesophyll. Despite the increases in A and gs, leaf transpiration (*E*) remained unchanged in all treatments, which, combined with the increase in A, resulted in substantial gains in water use efficiency (WUE), reaching 52% with Mg+AA. Carboxylation efficiency (*A/Ci*) responded similarly, with the Mg+AA treatment producing the greatest gain (66%), reinforcing the positive effect of combined foliar application on photosynthetic performance. Notably, both gs and E were higher in 2022, which is consistent with the greater water availability during the evaluation period (**Supplementary Table 3**; **Figure 1**).

### 3.5 Nitrate reductase activity

**Figure 5:**
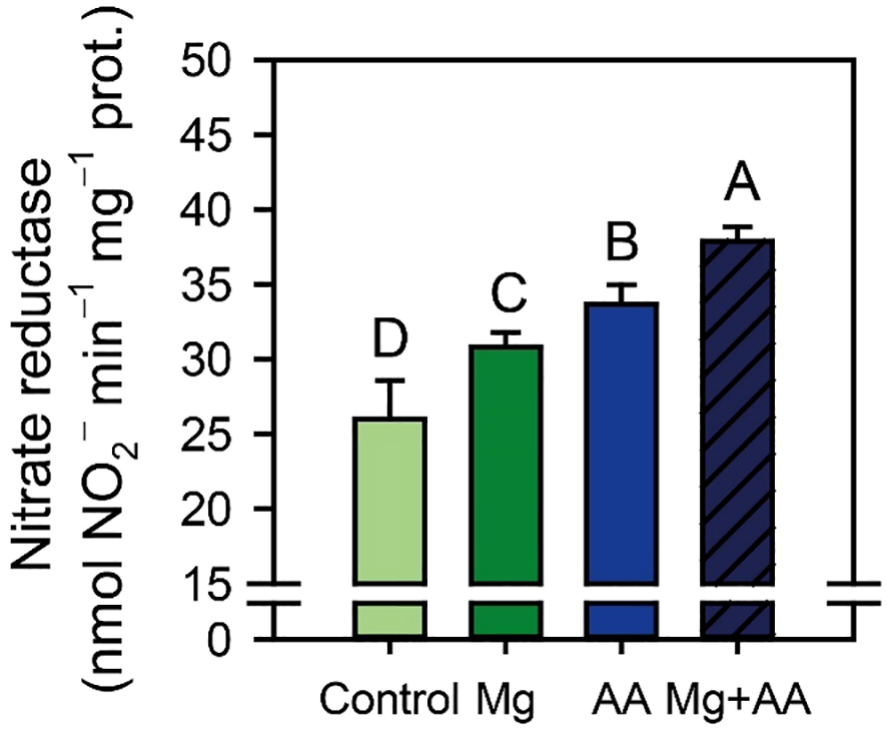
Nitrate reductase activity in the leaves of maize plants as affected by the foliar application of magnesium (Mg) and/or amino acids (AA). Bars represent means across the 2021 and 2022 off-season growing periods. Different letters indicate significant differences among treatments (LSD, p ≤ 0.05). Error bars represent standard deviation (n = 8).

All foliar applications of Mg and/or AA increased NR activity (**Figure 5**). Applying Mg, AA, and Mg+AA increased NR activity by 18%, 29%, and 45%, respectively, compared with the control.

### 3.6 Ribulose-1,5-bisphosphate carboxylase/oxygenase activity (Rubisco)

**Figure 6:**
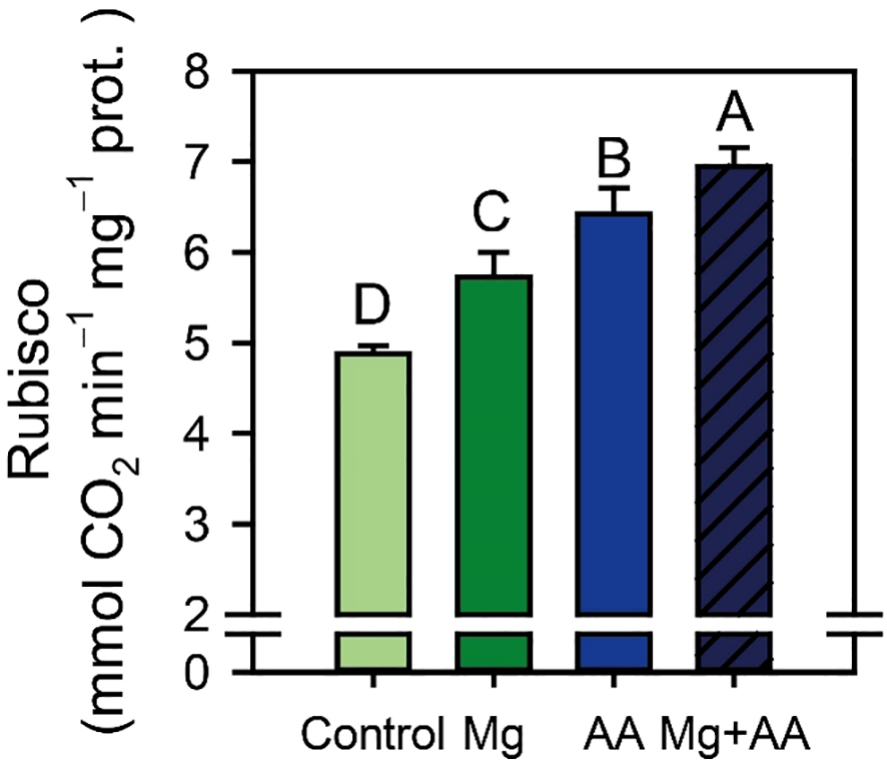
Rubisco activity in the leaves of maize plants as affected by the foliar application of magnesium (Mg) and/or amino acids (AA). Bars represent means across the 2021 and 2022 off-season growing periods. Different letters indicate significant differences among treatments (LSD, p ≤ 0.05).Error bars represent standard deviation (n = 8).

Rubisco activity increased under all foliar treatments and followed the same response pattern observed for gas exchange and nitrate reductase activity (**Figure 6**). The combined Mg+AA treatment promoted the strongest response (+42%), whereas Mg and AA resulted in intermediate increases (18%, 31% relative to the control). In addition, Rubisco activity was higher in 2022 than in 2021 (**Supplementary Table 4**).

### 3.7 ROS production and antioxidant enzyme activity

**Figure 7:**
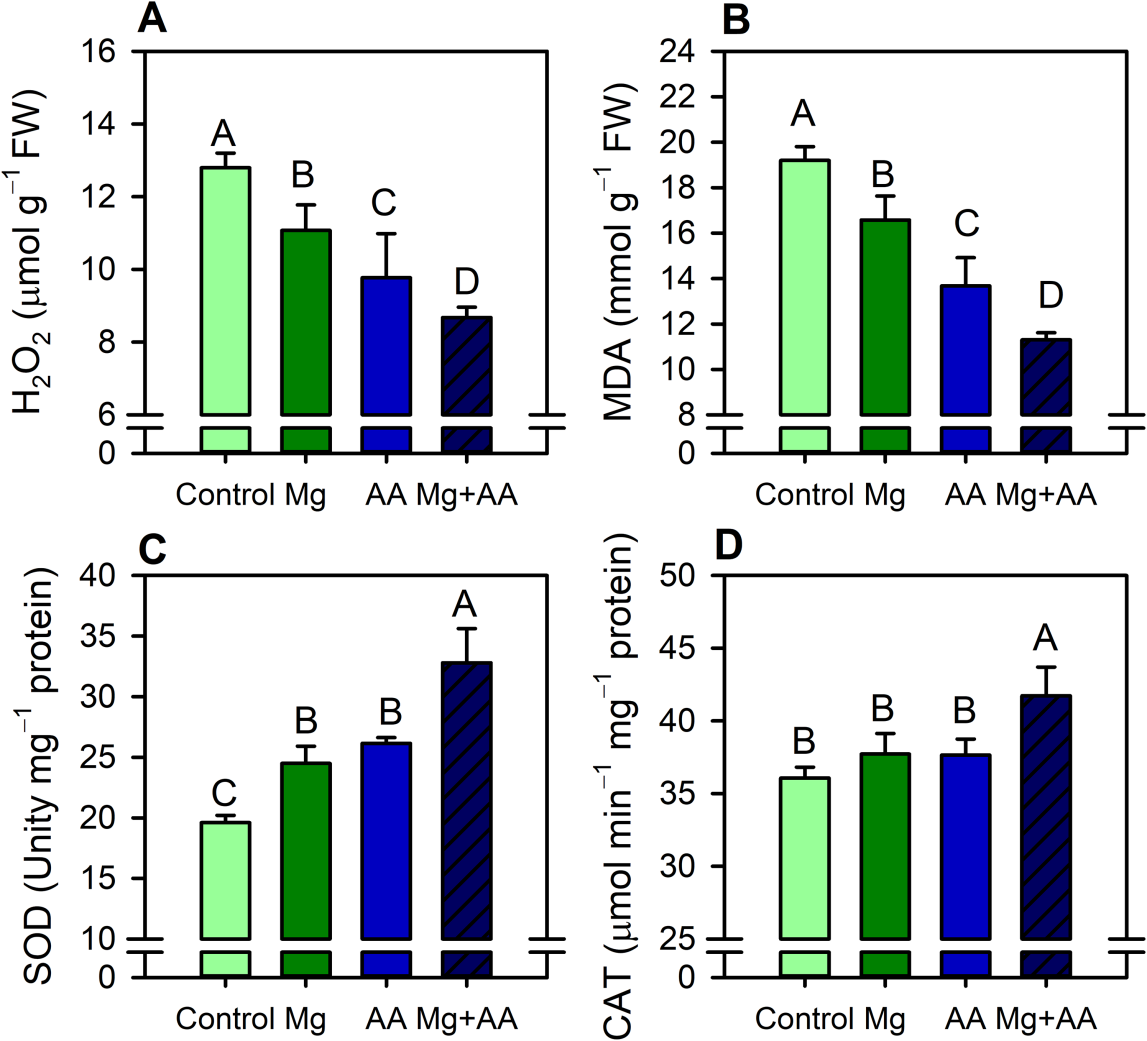
Concentrations of **(a)** hydrogen peroxide (H_2_O_2_), **(b)** malondialdehyde (MDA), **(c)** superoxide dismutase (SOD), and **(d)** catalase (CAT) in maize leaves as affected by the foliar application of magnesium (Mg) and/or amino acids. Bars represent the mean values from the 2021 and 2022 harvests conducted in Botucatu-SP, Brazil. Bars with different letters are significantly different according to the LSD test (*p* < 0.05). The error bars indicate the standard deviation (*n* = 8).

Foliar applications reduced ROS accumulation and enhanced antioxidant enzyme activity in maize leaves (**Figure 7**). Hydrogen peroxide (H_2_O_2_) concentration decreased by 14%, 24%, and 32% under Mg, AA, and Mg+AA applications, respectively, relative to the control. Similarly, malondialdehyde (MDA) concentration declined by 14%, 29%, and 41%, respectively, with the largest reduction observed under the combined treatment. In parallel, superoxide dismutase (SOD) activity increased substantially under foliar applications, reaching gains of 67% under Mg+AA, while catalase (CAT) activity also increased across treatments, particularly under the combined application. Lower ROS accumulation was observed in 2022, coinciding with higher precipitation; however, treatment responses remained consistent across growing seasons.

### 3.8 Grain yield and productivity components

**Figure 8:**
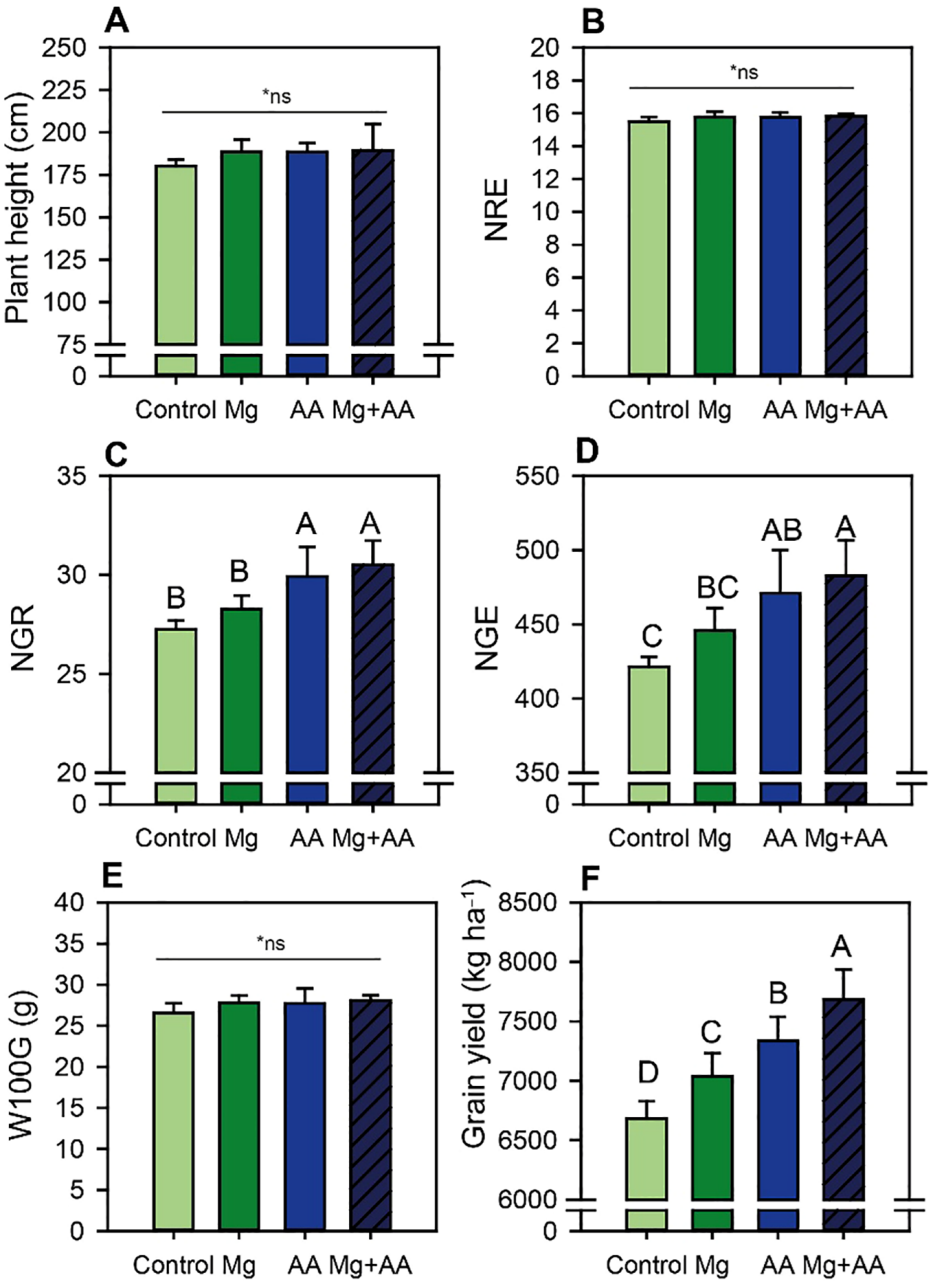
Effects of the foliar application of magnesium (Mg) and/or amino acids (AA) on **(a)** plant height and **(b-f)** production components: **(b)** number of rows per ear (NRE), **(c)** number of grains per row (NGR), **(d)** number of grains per ear (NGE), **(e)** hundred-grain weight (W100G), and **(f)** maize grain yield. Bars represent the mean values from the 2021 and 2022 harvests conducted in Botucatu-SP, Brazil. Bars with different letters are significantly different according to the LSD test (*p* < 0.05). *ns, not significant. The error bars indicate the standard deviation (*n* = 8).

Foliar applications of Mg and/or AA led to significant increases in grain yield and yield components (**Figure 8**). Plant height, number of rows per ear (NRE) and hundred-grain weight (W100G) did not differ among treatments, indicating that yield gains were driven by improved grain set rather than individual grain filling. Number of grains per row (NGR) and per ear (NGE) increased significantly under AA and Mg+AA, with NGE increasing by 15% relative to the control. The Mg+AA treatment produced the highest yield gain (1,001.7 kg ha^-^¹ above the control), followed by AA (654.8 kg ha^-^¹) and Mg (353.5 kg ha^-^¹). Although the growing season significantly influenced grain yield (an increase of 982 kg ha^-^¹ in the 2022 crop season compared to 2021), the responses to the treatments were consistent across both growing seasons, demonstrating their agronomic effectiveness.

### 3.9 Proteins in grain

**Figure 9:**
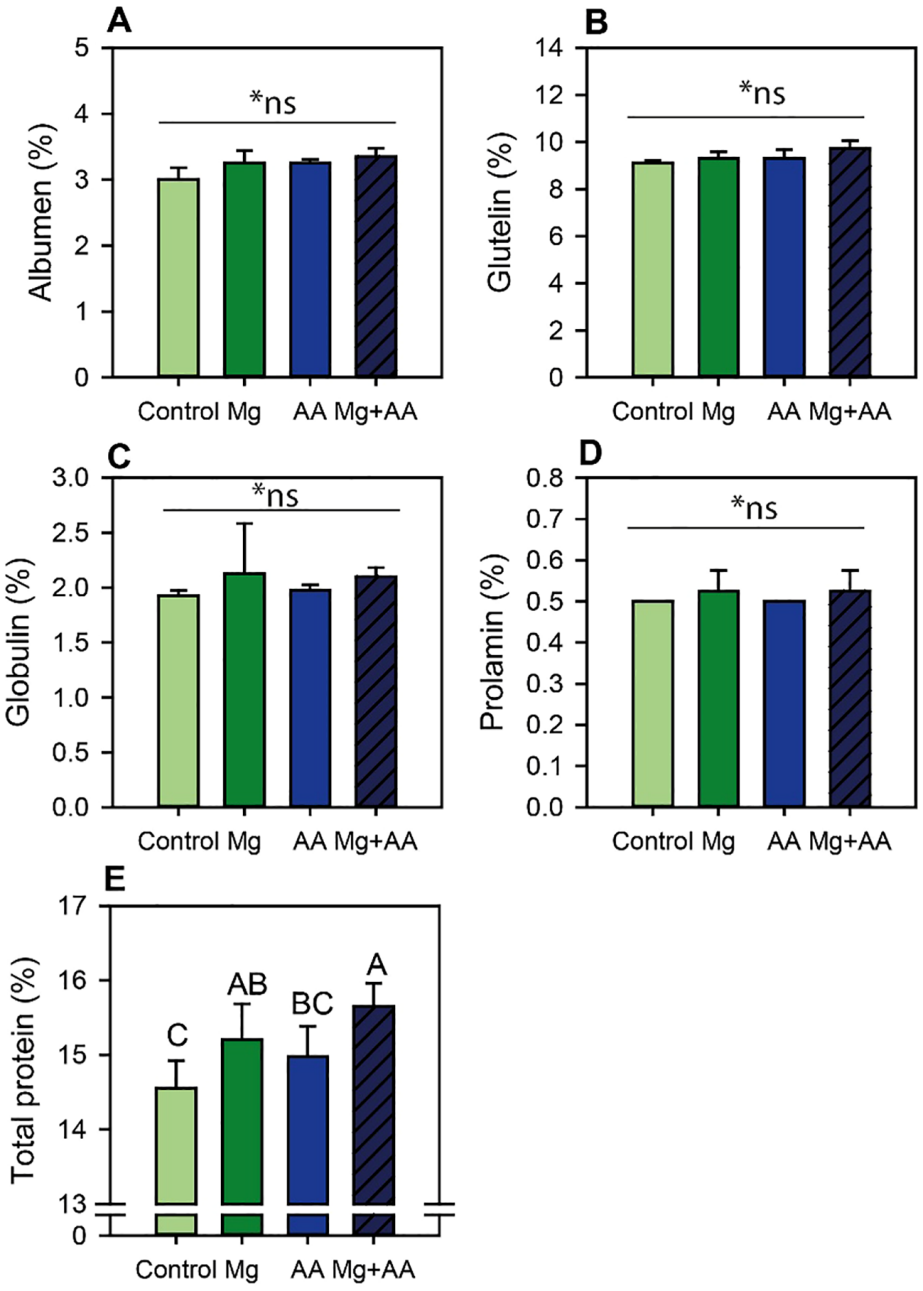
Concentrations of albumin **(A)**, glutelin **(B)**, globulin **(C)**, prolamin **(D)**, and total protein **(E)** in maize grain under the influence of the foliar application of magnesium (Mg) and/or amino acids (AA). The bars present the average values for the 2021 and 2022 harvests in Botucatu-SP, Brazil. Bars with different letters are significantly different according to the LSD test (p<0.05). The error bars indicate the standard deviation (n=8). *ns, not significant.

The treatments did not influence the individual protein content of the grains. However, the total protein content was affected by the applications of Mg and/or AA. (**Figure 9**). The average grain contents of albumin, glutelin, globulin, and prolamin were 3.2, 9.2, 2.0, and 0.5%, respectively. Despite these small individual increases, the foliar application of Mg and Mg+AA increased the total protein content of maize grain by 4.6% and 7.6%, respectively, compared with the control.

**Figure 10:**
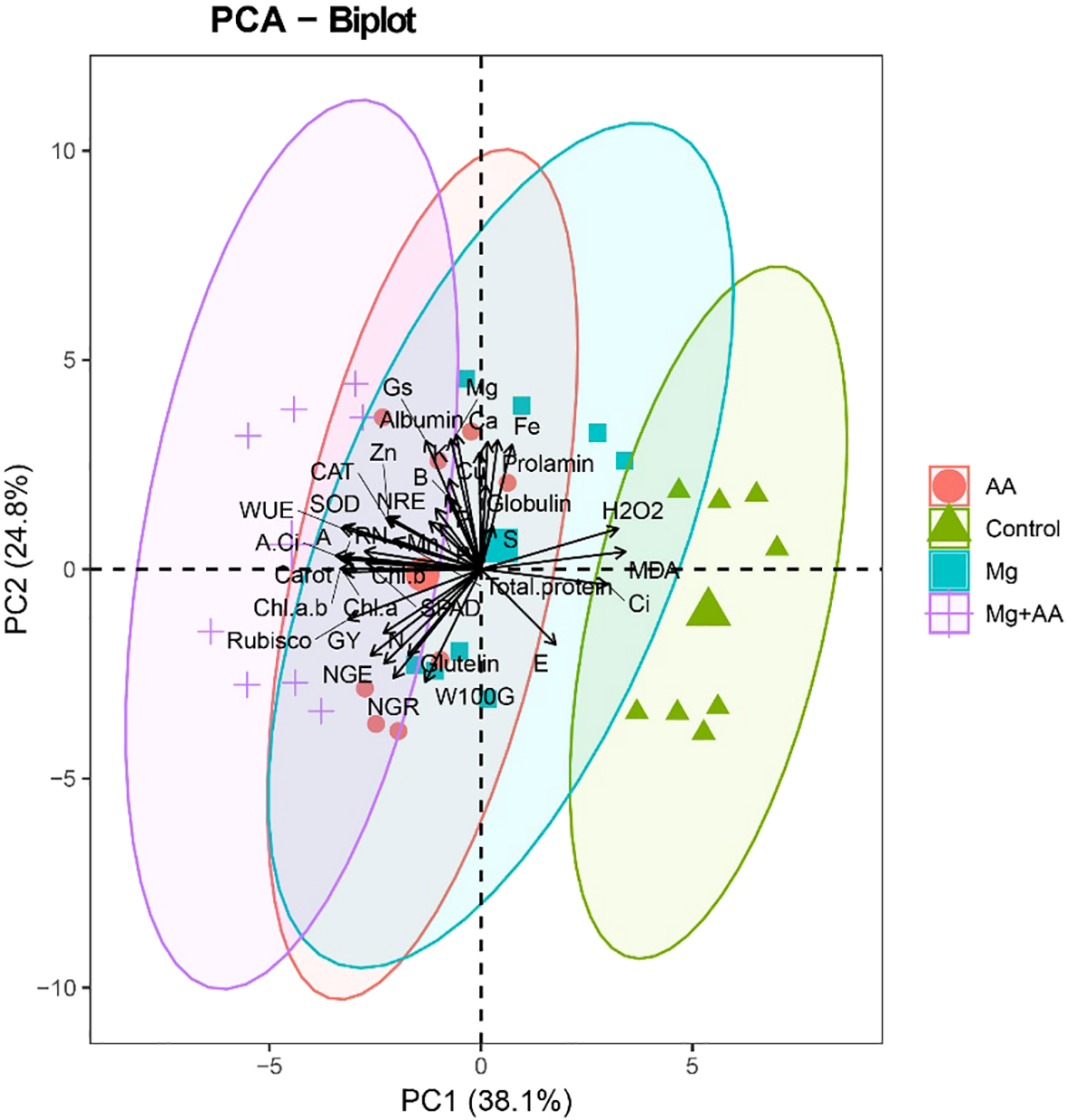
Principal component analysis (PCA) biplot of physiological, biochemical, nutritional, and yield variables of maize under Control, amino acids (AA), magnesium (Mg), and magnesium plus amino acids (Mg+AA) treatments. PC1 and PC2 explained 38.1% and 24.8% of the total variance, respectively. Ellipses represent the 95% confidence intervals of each treatment group.

The principal component analysis (PCA, **Figure 10**) explained 62.9% of the total variance, with PC1 and PC2 accounting for 38.1% and 24.8%, respectively. A clear separation among treatments was observed, indicating distinct physiological and biochemical responses. The Control treatment was associated with higher *Ci*, H_2_O_2_, and MDA levels, suggesting greater oxidative stress and lower photosynthetic efficiency. In contrast, the Mg+AA treatment was closely associated with antioxidant enzymes (CAT and SOD), stomatal conductance (*gs*), water use efficiency (WUE), Rubisco activity, and grain yield (GY), indicating enhanced physiological performance. The Mg treatment showed a stronger relationship with leaf Mg, Fe, and S concentrations, while AA alone exhibited an intermediate response. Overall, the PCA highlights the additive effect of Mg and amino acids in improving plant metabolism and productivity while reducing oxidative stress.

## 4 Discussion

Off-season maize cultivation is often threatened by adverse weather conditions, such as high temperatures and low water availability. In the present study, rainfall was absent for long periods in the off-season maize seasons in 2021 and 2022, resulting in water deficits at decisive stages for defining NGE and grain filling (**Figure 1**). This condition was especially severe in 2021, when the pronounced rainfall shortage led to reduced nitrogen uptake and decreased photosynthetic and antioxidant activities in maize plants. Our findings indicates that the foliar application of AA and Mg stimulates N, carbon (C), and antioxidant metabolism, thereby mitigating the physiological impacts of seasonal water deficits and supporting increased grain productivity under adverse climatic conditions.

Effects of magnesium and amino acids on macro- and micronutrient metabolism.

In the control, leaf Mg content was 2.8 g kg^-^¹, within the optimal range for maize [2.5 g kg^-^¹; Novais et al., 2007], hereas foliar application of Mg or Mg+AA increased it to 3.7 g kg^-^¹. The effectiveness of foliar Mg application is associated with its rapid absorption by leaves and its subsequent redistribution to metabolically active tissues. In maize, Mg exhibits intermediate phloem mobility and may be remobilized from vegetative tissues depending on source–sink demand and plant nutritional status (Chen et al., 2016; Altarugio et al., 2017).

Foliar Mg application rates ranging from 50 to 1,500 g Mg ha^-^¹ under Brazilian field conditions improved physiological performance and grain yield in maize, with maximum yield responses observed at rates between approximately 540 and 890 g Mg ha^-^¹ (Altarugio et al., 2017. Therefore, the 500 g Mg ha^-^¹ used in the present study represents a supplemental dose within the lower-to-intermediate agronomically effective range for maize. In this case, the application of AA contributed less to leaf Mg content, as the combined application of Mg and AA did not differ significantly from the isolated application of Mg. However, the isolated application of AA did increase Mg concentration in the leaves compared to the control.

Furthermore, although direct literature linking exogenous AA to enhanced Mg^2+^ uptake remains limited, the available data allow the inference that the combined treatment combined treatment (Mg+AA) significantly reduced H_2_O_2_ and MDA levels (**Figure 7**). This mitigation of oxidative stress likely mitigated lipid peroxidation, thereby preserving plasma membrane stability and protecting the membrane-bound transport systems responsible for nutrient uptake (Saxena et al., 2016; Cazar et al., 2023). Consequently, this inferred cellular protection may help explain why the supplemental foliar application of Mg was more effective when combined with AA.

The foliar application of Mg+AA also increased maize leaf nitrogen (N) content to the range considered adequate [30 g kg^−1^ (Novais et al., 2007)]. Mg functions in N metabolism by regulating the expression of the genes encoding glutamine synthetase isoenzymes (CsGSs), which catalyze the reaction of ammonium (NH_4_^+^) and glutamate to form glutamine and are essential for N assimilation by plants (Zhang et al., 2023). AA are an organic source of N, which may explain, at least in part, the increase in leaf N content in maize plants, as suggested by several authors (Jones, 2002; Vinall et al., 2012; Shanthi et al., 2018). Therefore, while the nutritional and signaling roles of the amino acid N cannot be entirely decoupled in the absence of an N-equivalent control, their combined action with Mg resulted in a clear additive and complementary enhancement of N assimilation pathways.

The effects of Mg and AA on N metabolism may be associated with nitrate reduction, an important N assimilation pathway. Nitrate reduction is catalyzed by NR, which converts nitrate to nitrite (Lu et al., 2016). In this study, the foliar application of Mg and/or AA increased NR activity. The foliar application of AA may increase NR activity through multiple mechanisms, such as promoting the synthesis of precursor proteins/enzymes, increasing the energy available for the activation of NR, or enhancing photosynthesis, as suggested by Teixeira et al. (2018). Furthermore, AA such as Cys, Arg, Lys, Ser, Tyr, and Asp are present in the structure of the molybdenum cofactor (MoCo) found in the active site of NR, where they stabilize MoCo or catalyze the reduction reaction (Coelho and Romão, 2015).

NR is activated by environmental factors such as the presence of nitrate, light, or CO_2_ (Bucher and Kossmann, 2007). The increase in NR activity under foliar Mg application may be related to downstream effects of Mg on ammonia (NH_3_) consumption through the activation of glutamate synthetase (Marschner, 2011), since NH_3_ accumulation negatively regulates NR activity (Kavoosi et al., 2014), or increased CO_2_ assimilation (Marschner, 2011).

The results also show that the foliar application of Mg has a greater effect on leaf P levels than does foliar AA application, as leaf P content did not differ between the Mg+AA and Mg treatments. Positive effects of Mg and P on root development have been proposed (Weih et al., 2021). It is possible that the increase in Mg content in maize plants favored the absorption or assimilation of P, however, further studies are needed to confirm this relationship.

### 4.1 Magnesium and amino acids act on different aspects of photosynthetic metabolism

In general, the foliar application of Mg and/or AA efficiently increased photosynthetic activity (**Figures 4**-**6**). Both biostimulants increased maize leaf levels of Chl *a*, Chl *b*, total Chl, and carotenoids. Mg is a structural component of chlorophyll (Ling et al., 2011), and AA such as Gly, Glu, and Ser are precursors in chlorophyll synthesis (Chatterjee and Kundu, 2015). Furthermore, the exogenous application of AA provides an organic source of N, which contributes to the synthesis of chlorophyll, as Mg is coordinated by four nitrogen atoms in the porphyrin ring (Taiz et al., 2018). While foliar application of Mg was effective, it is possible that the application of amino acids played a more significant quantitative role in the biosynthesis of chlorophyll compared to the foliar application of Mg.

In general, the SPAD index reflected the changes in photosynthetic pigment levels. The SPAD index is positively correlated with leaf chlorophyll and N contents and is a practical, cost-efficient and non-destructive evaluation method (Ling et al., 2011). Despite this, SPAD readings are less accurate than laboratory methods and the output readings are unitless leaf chlorophyll content, requiring an association with the laboratory method to attest to the accuracy of the results (Zhang et al., 2022).

Foliar Mg and/or AA supplementation also influenced photosynthetic parameters, with increases in *gs* and decreases in *Ci*. Increases in *gs* enhance the concentration of CO_2_ that can diffuse into the stomata. The reduction in *Ci* and increase in Rubisco activity indicate that CO_2_ was effectively assimilated, culminating in greater *A*. An increase in *A* in combination with a decrease in *Ci* increases *A/Ci*. Electron transport rates are typically influenced by chlorophyll abundance (Perchlik and Tegeder, 2018). Furthermore, AA such as Ala, Gly, and Ser participate in the active site of Rubisco (Allen et al., 2012), and the activation of these residues is regulated by an essential Lys residue that is stabilized by a Mg ion to form a catalytically active complex (Portis and Parry, 2007). Thus, on the basis of the observed increases in chlorophyll content, Rubisco activity, and *A*, we infer that the foliar application of Mg and AA, especially when applied simultaneously, improves both the photochemical and biochemical components of photosynthesis.

Despite the increase in *gs* under treatments with Mg and/or AA compared to the control, leaf transpiration (*E*) remained constant. This result is unexpected, as an increase in *gs* typically correlates with a rise in *E* (Guerrieri et al., 2019). However, environmental and structural factors—such as the canopy boundary layer, local humidity around the leaves, adjustments in photosynthesis and CO_2_ accumulation— can stabilize *E* even when *gs* is changed (Temple, 1986; Meinzer and Grantz, 1989; McNaughton and Jarvis, 1991). The stability in *E* and increase in *A*, were associated with greater instantaneous (leaf-level) WUE, indicating that the plant was able to increase CO_2_ assimilation without increasing water loss. This result is particularly important and suggests that the foliar application of Mg and AA can improve the tolerance of maize plants to water deficit, which occurred during critical phenological stages for defining productivity.

### 4.2 Effects of foliar magnesium and amino acid application on antioxidant activity

Studies of the exogenous supply of AA to plants have explored antioxidant effects (Teixeira et al., 2017; Haghighi et al., 2022). A previous study demonstrated that foliar Mg application efficiently improves the antioxidant metabolism of soybean and maize plants (Rodrigues et al., 2021a). The present study investigated the stress-mitigating effects of the combined application of AA and Mg on off-season maize. The results corroborate our hypothesis, as the reductions in H_2_O_2_ and MDA levels were greatest when AA and Mg were applied together. The decreases in these ROS were associated with increases in the activities of the antioxidant enzymes SOD and CAT. Although the foliar application of either AA or Mg significantly increased SOD activity, the combined application of these biostimulants further enhanced SOD activity. CAT activity was less responsive to the foliar application of AA and Mg; a significant increase in CAT activity (p<0.05) was observed only in the Mg+AA treatment.

SOD is the first line of defense against ROS and catalyzes the conversion of O_2_^–^ to H_2_O_2_ and O_2_, more stable ROS. CAT catalyzes the conversion of H_2_O_2_ to H_2_O and O_2_ (Gill et al., 2015; Stephenie et al., 2020) (Gill et al., 2015; Stephenie et al., 2020) (Gill et al., 2015; Stephenie et al., 2020). The combined activity of these enzymes is essential to prevent the formation of other ROS that are more harmful to cellular components. The reduction in the maize leaf content of MDA, a product of lipid peroxidation and an indicator of cellular injury (Grotto et al., 2008) (Grotto et al., 2008) (Grotto et al., 2008), corroborates the effectiveness of SOD and CAT at preventing toxic levels of ROS.

Under water deficit conditions, plants close their stomata to prevent water loss, but this closure hinders the entry of CO_2_ for reduction. This situation is aggravated under limited Mg, which impairs phloem loading, because sucrose accumulation negatively regulates CO_2_ assimilation and reduces NADP^+^ regeneration capacity. Without efficient regeneration of electron sinks, excessive reduction of the electron transport chain leads to photoreduction of O_2_ to O_2_^–^ (Cruz de Carvalho, 2008; Tränkner et al., 2018). Additionally, exogenous Mg may play a role in activating antioxidant enzymes, protecting cellular structures and functions, and promoting osmotic adjustment and maintenance of cellular turgor under stressful conditions (Khanchi et al., 2024). Although direct biophysical indicators of plant water status (e.g., leaf water potential or relative water content) were not evaluated in this study, the distinct mitigation of oxidative stress markers (such as H_2_O_2_ and MDA levels) and the upregulation of the antioxidant enzyme machinery strongly indicate a downstream alleviation of drought-induced metabolic disorders. The absence of these biophysical parameters represents a limitation of the current evaluation, and further research is warranted to fully characterize the systemic mechanisms of water stress mitigation.

AA are important components of plant antioxidant metabolism and are involved in the reduction of free radicals (Teixeira et al., 2017). For example, Glycine supplementation induces the production of glycine betaine, which functions in osmoprotection and antioxidant signaling, and glyoxylate, which is involved in the reduction of H_2_O_2_ and production of NADPH and ATP during photosynthesis (Hu et al., 2012; Alhasawi et al., 2015). Cysteine supplementation efficiently reduces free radicals and increases the activities of CAT and phenylalanine ammonia-lyase (PAL), an important enzyme of secondary metabolism that utilizes phenylalanine as a substrate (Azarakhsh et al., 2015). Glutamate is involved in the synthesis of proline and arginine, which are involved in osmoprotection (Gill and Tuteja, 2010). Thus, the antioxidant effects of foliar application of AA can be attributed to its influence on the antioxidant machinery. When combined with Mg’s role in enzymatic activation and cellular protection, this response strengthens the overall antioxidant action in maize plants. Furthermore, a non-enzymatic pathway, such as the osmoprotective role induced by Mg and AA, may explain the reduction in H_2_O_2_ and MDA concentrations even when SOD and CAT enzymes were not altered (**Figure 7**).

The observed enhancement of antioxidant enzyme activities and the reduction in oxidative damage markers are also in agreement with previous studies reporting stress-mitigating effects of foliar Mg or AA supply (Rodrigues et al., 2021a; Haghighi et al., 2022). However, the greater reductions in H_2_O_2_ and MDA levels under the combined Mg+AA treatment suggest an additive effect, which has been less frequently reported and highlights the novelty of the present findings.

### 4.3 Grain yield and grain protein content

Water deficits during flowering negatively affect fertilization by causing sterility of the pollen grain, limiting ear differentiation, suppressing the elongation of silks, and, after fertilization, inducing grain abortion. Sucrose limitation is one of the main factors leading to grain abortion because it increases competition for photoassimilates between sibling grains (Shen et al., 2020) (Shen et al., 2020) (Shen et al., 2020). In addition, low sucrose synthase activity reduces pollen grain viability, which is positively correlated with the concentrations of carbohydrates and their derivatives (Li et al., 2022).

In the present study, the foliar application of Mg and/or AA increased the number of grains per row (NGR) and per ear (NGE), even under water deficit conditions during flowering. These yield responses can be directly linked to the improvements observed in leaf nutritional status, photosynthetic performance, and antioxidant capacity. Previous studies support a direct relationship between improved antioxidant and photosynthetic activity as a mechanism for increasing grain productivity (Rodrigues et al., 2021a, 2021b; Galeriani et al., 2022; Jacomassi et al., 2022; Oliveira et al., 2022; de Souza et al., 2025). Enhanced chlorophyll content, Rubisco activity, CO_2_ assimilation (*A*), and instantaneous water use efficiency indicate a greater capacity for carbon fixation without proportional increases in water loss. Simultaneously, the enhanced antioxidant system reduced oxidative damage, preserving photosynthetic integrity during a critical phenological stage. Together, these physiological adjustments likely sustained carbohydrate supply to reproductive organs, favoring kernel set and reducing grain abortion, ultimately resulting in higher grain yield.

In general, the physiological effects of foliar AA application were greater than those of foliar Mg application. However, the best results were obtained when both products were applied. The magnitude of yield increase observed in the present study (~15%) is comparable to or slightly higher than the average yield gains reported for foliar Mg application alone (~10%; Altarugio et al., 2017; Rodrigues et al., 2021a), but lower than the maximum increases reported for amino acid-based biostimulants under optimal conditions (Brankov et al., 2020; Ebaid et al., 2025). Part of this variation may be attributed to seasonal environmental conditions. These discrepancies likely reflect differences in environmental conditions, particularly the occurrence of water deficit during flowering in the present study, which constrained the full expression of yield potential.

Although the treatment × season interaction was not significant, the greater grain yield observed in the 2022 season was accompanied by higher stomatal conductance (*gs*) and transpiration (E), greater chlorophyll accumulation, and higher Rubisco activity, indicating enhanced gas exchange and photosynthetic performance under the environmental conditions of that year. In addition, the lower H_2_O_2_ and MDA concentrations observed in 2022 indicate reduced oxidative damage, which was associated with improvements in key yield components such as NGE and W100G. Together, these responses suggest that the environmental conditions in 2022 favored greater physiological activity, more efficient carbon assimilation, and improved assimilate allocation to reproductive structures, ultimately contributing to the higher grain yield observed in that season. Within this environmental context, the combined application of Mg and AA appears to be more effective than isolated applications, as it simultaneously supports photosynthetic efficiency, antioxidant protection, and nutrient assimilation.

Despite the enhancement of photosynthetic and antioxidant metabolism and the increase in grain yield under Mg and/or AA application, no significant differences were observed in the concentration of individual grain proteins. Considering that total protein content in maize grains typically ranges from approximately 8 to 15% (Langyan et al., 2021; Fan et al., 2024; Yin et al., 2025), as observed in this study, the detection of small changes in individual proteins was limited. However, these minor increases collectively resulted in a significant increase in total grain protein content, particularly in the Mg+AA treatment.

Grain protein content tends to be responsive to increases in N supply to the plant (Wan et al., 2023). In this context, the higher leaf N content, increased nitrate reductase activity, and enhanced N assimilation observed under Mg and AA application likely supported greater N remobilization to the grains during filling. Consequently, the combined foliar application of Mg and AA not only increased grain yield but also improved total grain protein content, enhancing the nutritional value of maize for human and animal consumption (Guimarães and Pacheco, 1998).

The multivariate analysis further reinforced these relationships. The PCA revealed a clear separation among treatments, corroborating the physiological, biochemical, and nutritional responses observed throughout the study. The Mg+AA treatment was closely associated with variables related to photosynthetic performance, antioxidant metabolism, nutrient assimilation, and grain yield, indicating a coordinated improvement in plant functioning under the environmental conditions of the off-season. In contrast, the Control treatment was associated with H_2_O_2_ and MDA, reflecting greater oxidative damage and reduced physiological performance. The AA and Mg treatments occupied intermediate positions, suggesting partial mitigation of these effects when applied individually. Overall, the PCA supports the hypothesis that Mg and amino acids act through complementary mechanisms that enhance nutrient use, antioxidant protection, carbon assimilation, and ultimately grain productivity.

## 5 Conclusions

The foliar application of Mg and AA efficiently improved the nutritional status, photosynthetic activity and antioxidant metabolism of off-season maize under water deficit conditions. The combined application of Mg and AA demonstrated additive effects, leading to greater enhancements in nutrient assimilation, photosynthetic efficiency, and antioxidant defense compared to isolated applications. These physiological improvements resulted in a 15% increase in grain yield and higher total protein content in the grains under the Mg+AA treatment. The study highlights the complementary roles of Mg and AA in mitigating stress and enhancing productivity, with Mg supporting chlorophyll synthesis and enzyme activation, while AA acts as organic nitrogen sources and stress-mitigating agents. These findings suggest that the combined foliar application of Mg and AA is a promising strategy to improve maize resilience and yield under challenging environmental conditions.

## Data Availability

The original contributions presented in the study are included in the article/**Supplementary Material**, further inquiries can be directed to the corresponding author/s.
