# Supplementary material for: Foliar application of magnesium and amino acids to off-season maize: a strategy to improve photosynthetic and antioxidant metabolism
Source: Front Plant Sci. 2026 Jul 1;17:1850949. doi: 10.3389/fpls.2026.1850949 (PMC13368494; doi:10.3389/fpls.2026.1850949)
Supplement: Supplementary file 1 [file Table1.docx]

**Supplementary material**

**Table S1**. Physical and chemical characteristics of the soil in the experimental area. Botucatu – SP, 2021

| Soil physical characteristics | | | | | | | |
| --- | --- | --- | --- | --- | --- | --- | --- |
| Clay | | Silt | | Sand | | Density | |
| .................................... g kg^−1^ .................................... | | | | | | .......... g cm^-3^ .......... | |
| 602.0 | | 281.0 | | 117.0 | | 1.19 | |
| Soil chemical characteristics | | | | | | | |
| pH (CaCl_2_) | M.O | P (resin) | Ca^2+^ | Mg | K^+^ | Al^3+^ | Potential acidity |
| -- | g dm^3^ | mg dm^3^ | .................................... mmol_c_ dm^3^ .................................... | | | | |
| 5.0 | 30.4 | 29.6 | 37.6 | 13.7 | 1.6 | 1.5 | 32.5 |
| S | B | Cu | Fe | Mn | Zn | SC | CEC _pH 7,0_ |
| ........................................... mg dm^3^................................................. | | | | | | (%) | mmol_c_ dm^3^ |
| 4.90 | 0.5 | 6.0 | 22.0 | 19.2 | 1.2 | 60.6 | 85.3 |

Ca^2+^, Mg, K^+^ were determined by the resin method; Al^3+^ was determined by KCl extraction; B was determined by hot water extraction, Cu, Fe, Mn, and Zn were determined by extraction in DTPA solution

**Table S2.** Macro- and micronutrient contents, photosynthetic pigment contents, and gas-exchange parameters of second-crop maize under the influence of the foliar application of magnesium (Mg) and/or amino acids (AA) in two growing seasons, 2021 and 2022. Botucatu, SP, Brazil.

|  | N | P | K | Ca | Mg | S | Fe | Cu | Zn | Mn | B |
| --- | --- | --- | --- | --- | --- | --- | --- | --- | --- | --- | --- |
| *Year* |  | | | | | |  | | | | |
| 2021 | 29.8 b | 3.1 a | 23.4 a | 4.4 a | 4.0 a | 1.6 | 156.6 a | 8.6 a | 20.4 | 39.9 | 10.7 a |
| 2022 | 35.0 a | 2.7 b | 19.5 b | 3.2 b | 2.6 b | 1.5 | 105.0 b | 6.2 b | 19.5 | 39.1 | 9.4 b |
| *Treatments* |  |  |  |  |  |  |  |  |  |  |  |
| **2021** | | | | | | | | | | | |
| Control | 25.8 b | 2.9 | 20.8 | 4.3 | 3.3 c | 1.6 | 152.5 | 7.7 | 17.3 | 37.2 | 9.3 |
| Mg | 31.6 a | 3.2 | 23.8 | 4.5 | 4.6 a | 1.7 | 155.0 | 8.7 | 20.4 | 40.8 | 11.6 |
| AA | 31.5 a | 3.0 | 24.4 | 4.5 | 3.9 b | 1.6 | 157.8 | 9.3 | 21.6 | 40.8 | 10.5 |
| Mg+AA | 30.2 a | 3.4 | 24.6 | 4.4 | 4.3 ab | 1.6 | 161.3 | 8.9 | 22.4 | 40.7 | 11.4 |
| **2022** | | | | | | | | | | | |
| Control | 30.1 b | 2.4 c | 17.9 | 2.9 | 2.3 c | 1.5 | 106.0 | 6.2 | 17.5 | 38.3 | 8.5 |
| Mg | 36.0 a | 3.0 a | 20.3 | 3.4 | 2.8 b | 1.6 | 100.3 | 6.1 | 20.0 | 39.0 | 9.5 |
| AA | 35.5 a | 2.6 bc | 20.4 | 3.3 | 2.5 c | 1.5 | 107.1 | 6.6 | 20.3 | 39.6 | 10.0 |
| Mg+AA | 38.3 a | 2.8 ab | 19.5 | 3.2 | 3.0 a | 1.5 | 106.6 | 6.1 | 20.4 | 39.4 | 9.5 |
|  |  |  |  |  |  |  |  |  |  |  |  |
| *F probability* |  |  |  |  |  |  |  |  |  |  |  |
| Year (Y) | <0.0001 | 0.0194 | 0.0072 | <0.0001 | <0.0001 | 0.2671 | <0.0001 | <0.0001 | 0.3507 | 0.5096 | 0.0334 |
| Treatments (T) | 0.006 | 0.0013 | 0.3494 | 0.4325 | <0.0001 | 0.6164 | 0.8966 | 0.1962 | 0.245 | 0.4291 | 0.1853 |
| Y x T | 0.4709 | 0.8518 | 0.9439 | 0.9668 | 0.0576 | 0.9978 | 0.966 | 0.4208 | 0.853 | 0.8241 | 0.7046 |

In g kg^−1^: Nitrogen (N), Phosphorus (P), Potassium (K), Calcium (Ca), Magnesium (Mg), Sulfur (S); in mg kg^−1^: Iron (Fe), Copper (Cu), Zinc (Zn), Manganese (Mn), Boron (B). Means followed by different letters are significantly different according to the F test (p<0.05).

Table S3. Photosynthetic pigment contents, and gas-exchange parameters of second-crop maize under the influence of the foliar application of magnesium (Mg) and/or amino acids (AA) in two growing seasons, 2021 and 2022. Botucatu, SP, Brazil.

| *Year* | Chl a | Chl b | Chl a+b | Carot | SPAD | *A* | *gs* | *Ci* | *E* | WUE | A/Ci |
| --- | --- | --- | --- | --- | --- | --- | --- | --- | --- | --- | --- |
|  |  | | | |  |  |  |  |  |  |  |
| 2021 | 1289 b | 362 | 1651 b | 330 | 30.2 | 21.4 | 137.2 b | 250.8 | 5.2 b | 4.2 | 0.0869 |
| 2022 | 1352 a | 365 | 1717 a | 339 | 30.8 | 22.1 | 198.2 a | 250.8 | 5.5 a | 4.1 | 0.0895 |
| *Treatments* |  |  |  |  |  |  |  |  |  |  |  |
|  | **2021** | | | | | | | | | | |
| Control | 1083 c | 326 b | 1409 c | 287 b | 27.5 b | 18.3 c | 114 c | 275 a | 5.5 | 3.3 c | 0.067 c |
| Mg | 1283 b | 348 b | 1631 b | 306 b | 29.4 b | 21.3 b | 131 bc | 261 ab | 5.2 | 4.1 b | 0.082 b |
| AA | 1348 ab | 388 a | 1736 ab | 357 a | 30.4 ab | 22.3 ab | 144 ab | 242 bc | 5.2 | 4.3 b | 0.092 b |
| Mg+AA | 1441 a | 388 a | 1829 a | 369 a | 33.4 a | 23.9 a | 159 a | 225 c | 4.8 | 5.0 a | 0.106 a |
|  | **2022** | | | | | | | | | | |
| Control | 1101 c | 331 c | 1432 c | 291 c | 27.9 c | 17.9 c | 166 b | 280 a | 5.6 | 3.2 c | 0.064 d |
| Mg | 1302 b | 391 a | 1693 b | 344 b | 33.0 a | 22.0 b | 197 ab | 251 b | 5.4 | 4.1 b | 0.088 c |
| AA | 1467 a | 373 ab | 1840 a | 329 b | 31.5 ab | 22.8 b | 206 a | 238 b | 5.5 | 4.2 b | 0.096 b |
| Mg+AA | 1540 a | 365 b | 1905 a | 392 a | 30.8 b | 25.8 a | 224 a | 235 b | 5.3 | 4.9 a | 0.110 a |
| *F probability* |  |  |  |  |  |  |  |  |  |  |  |
| Year (Y) | 0.0377 | 0.6183 | 0.0319 | 0.1596 | 0.2430 | 0.1326 | <0.0001 | 0.9935 | 0.0334 | 0.4041 | 0.2785 |
| Treatments (T) | 0.0001 | <0.0001 | 0.0001 | 0.0001 | <0.0001 | <0.0001 | <0.0001 | <0.0001 | 0.0589 | <0.0001 | <0.0001 |
| Y x T | 0.4804 | 0. 1900 | 0.7987 | 0.108 | 0.077 | 0.279 | 0.8078 | 0.5322 | 0.5676 | 0.9952 | 0.5582 |

In µg cm^2^: Chlorophyll a (Chl a), Chlorophyll b (Chl b), Chlorophyll a+b (Chl a+b), Carotenoids (Carot), SPAD index (SPAD), Net photosynthesis (*A*, µmol CO_2_ m^−2^ s^−1^), Stomatal conductance (*gs*, mmol H_2_O m^−2^ s^−1^), CO_2_ concentration in the substomatal chamber (*Ci*, µmol CO_2_ m^−2^ s^−1^), Transpiration rate (*E*, mmol H_2_O m^−2^ s^−1^), Water use efficiency (WUE, μmol CO_2_ (mmol H_2_O)^−1^), Carboxylation efficiency (*A*/*Ci*). Means followed by different letters are significantly different according to the F test (p<0.05).

**Table S4.** Enzymatic activity and biometric variables in second-crop maize under the influence of the foliar application of magnesium (Mg) and/or amino acids (AA) in two growing seasons, 2021 and 2022. Botucatu, SP, Brazil.

| *Year* | Rubisco | NR | H_2_O_2_ | MDA | SOD | CAT | Prolificacy | PH | NRE |  |
| --- | --- | --- | --- | --- | --- | --- | --- | --- | --- | --- |
| Year |  |  |  |  |  |  |  |  |  |  |
| 2021 | 5.51 b | 31.61 b | 11.41 a | 16.15 a | 26.29 | 38.98 | 0.99 | 189.7 | 15.9 |  |
| 2022 | 6.46 a | 32.55 a | 9.74 b | 14.22 b | 25.23 | 37.6 | 0.98 | 183.6 | 15.6 |  |
| *Treatments* |  |  |  |  |  |  |  |  |  |  |
|  | **2021** | | | | | | | | |  |
| Control | 4.45 d | 25.14 c | 13.05 a | 19.57 a | 20.01 c | 36.38 b | 1.00 | 182 | 15.7 |  |
| Mg | 4.98 c | 30.96 b | 12.28 ab | 18.42 a | 24.60 b | 38.41 ab | 1.00 | 192 | 15.9 |  |
| AA | 5.87 b | 33.10 b | 10.90 bc | 14.35 b | 27.28 b | 38.67 ab | 0.98 | 193 | 15.9 |  |
| Mg+AA | 6.75 a | 37.23 a | 9.43 c | 12.26 c | 33.26 a | 42.46 a | 0.99 | 192 | 15.9 |  |
|  | **2022** | | | | | | | | |  |
| Control | 5.30 c | 26.85 c | 12.57 a | 18.85 a | 19.18 c | 35.75 b | 1.00 | 179 | 15.3 |  |
| Mg | 6.50 b | 30.59 bc | 9.84 b | 14.75 b | 24.44 b | 37.02 b | 0.99 | 185 | 15.7 |  |
| AA | 6.93 a | 34.21 b | 8.65 bc | 12.98 b | 25.01 b | 36.65 b | 0.98 | 183 | 15.6 |  |
| Mg+AA | 7.12 a | 38.54 a | 7.91 c | 10.28 c | 32.30 a | 40.98 a | 0.98 | 187 | 15.8 |  |
|  |  |  |  |  |  |  |  |  |  |  |
| *F probability* | |  |  |  |  |  |  |  |  |  |
| Year (Y) | <0.0001 | 0.2054 | <0.0001 | 0.0017 | 0.1965 | 0.1587 | 0.7679 | 0.1181 | 0.048 |  |
| Treatments (T) | <0.0001 | <0.0001 | <0.0001 | <0.0001 | <0.0001 | 0.0027 | 0.5837 | 0.3139 | 0.3091 |  |
| Y x T | 0.115 | 0.7548 | 0.2146 | 0.2746 | 0.818 | 0.9637 | 0.9924 | 0.9258 | 0.9073 |  |

Rubisco activity (mmol CO_2_ min^−1^ mg^−1^ prot), Nitrate reductase activity (mmol NO_2_^−^ min^−1^ mg^−1^ prot), Hydrogen peroxide content (H_2_O_2_, µmol g^−1^ FW), Malondialdehyde content (MDA, mmol g^−1^ FW), Superoxide dismutase activity (SOD, unit mg^−1^ prot), Catalase activity (CAT, mmol min^−1^ mg^−1^ prot), Prolificacy (number of ears plant^-1^), Plant height (PH, cm), Number of rows per ear (NRE). Means followed by different letters are significantly different according to the F test (p<0.05).

**Table S5.** Productivity components, grain yield, and grain protein content in second-crop maize under the influence of the foliar application of magnesium (Mg) and/or amino acids (AA) in two growing seasons, 2021 and 2022. Botucatu, SP, Brazil.

| Treatments | NGR | NGE | W100G | GY | Albumin | Glutelin | Globulin | Prolamin | TP |
| --- | --- | --- | --- | --- | --- | --- | --- | --- | --- |
| Year |  |  |  |  |  |  |  |  |  |
| 2021 | 26.6 | 421.5 b | 25.3 b | 6693 b | 3.473 a | 8.891 b | 2.177 a | 0.587 a | 15.13 |
| 2022 | 31.4 | 489.1 a | 29.7 a | 7675 a | 2.937 b | 9.798 a | 1.854 b | 0.474 b | 15.06 |
|  |  |  |  |  |  |  |  |  |  |
| Control | 25 b | 389 b | 25 | 6235 c | 3.190 | 8.625 | 2.095 | 0.590 | 14.50 b |
| Mg | 26 ab | 416 ab | 26 | 6535 bc | 3.577 | 8.859 | 2.269 | 0.608 | 15.31 ab |
| AA | 28 a | 439 a | 25 | 6809 b | 3.500 | 8.772 | 2.128 | 0.568 | 14.97 ab |
| Mg+AA | 28 a | 442 a | 25 | 7193 a | 3.626 | 9.307 | 2.215 | 0.583 | 15.73 a |
|  |  |  |  |  |  |  |  |  |  |
| Control | 30 b | 454 c | 28 | 7128 c | 2.810 | 9.559 | 1.745 | 0.476 | 14.59 b |
| Mg | 30 b | 476 bc | 30 | 7535 bc | 2.917 | 9.736 | 1.979 | 0.488 | 15.12 ab |
| AA | 32 ab | 503 ab | 30 | 7864 ab | 2.981 | 9.803 | 1.753 | 0.451 | 14.99 ab |
| Mg+AA | 33 a | 523 a | 31 | 8173 a | 3.041 | 10.095 | 1.939 | 0.482 | 15.56 a |
|  |  |  |  |  |  |  |  |  |  |
| *F probability* | |  |  |  |  |  |  |  |  |
| Year (Y) | 0.0001 | <0.0001 | <0.0001 | <0.0001 | <0.0001 | <0.0001 | 0.0005 | <0.0001 | 0.7906 |
| Treatments (T) | 0.0013 | 0.0011 | 0.4252 | <0.0001 | 0.226 | 0.1633 | 0.2204 | 0.3299 | 0.0097 |
| Y x T | 0.9087 | 0.8676 | 0.3128 | 0.9469 | 0.5743 | 0.9755 | 0.9634 | 0.9847 | 0.9653 |

Number of grains per row (NGR), Number of grains per ear (NGE), Weight of one hundred grains (W100G, g), Grain yield (GY, kg ha^−1^), Albumin content in grain (mg g^−1^ DM), Glutelin content in grain (mg g^−1^ DM), Globulin content in grain (mg g^−1^ DM), Prolamin content in grain (mg g^−1^ DM), Total protein content in grain (mg g^−1^ DM). Means followed by different letters are significantly different according to the F test (p<0.05).
